# Unilateral pitting edema of the leg as a manifestation of Graves’ disease: a case report

**DOI:** 10.1186/1752-1947-6-258

**Published:** 2012-08-30

**Authors:** Vallo Volke, Svetlana Matjus

**Affiliations:** 1 Internal Medicine Clinic, South Estonian Hospital, Meegomäe, Võru vald, 65526, Estonia; 2 Endocrinology Unit, Tartu UniversityHospital, Puusepa 6, Tartu, 51014, Estonia; 3 Department of Physiology, Tartu University, Ravila 19, Tartu, 50411, Estonia

**Keywords:** Graves’ disease, Hyperthyroidism, Edema

## Abstract

**Introduction:**

Graves’ hyperthyroidism has a number of well-recognized but relatively rare extrathyroid manifestations such as thyroid acropachy, pretibial myxedema, and congestive heart failure.

**Case presentation:**

A 38-year-old Caucasian woman presented to the out-patient clinic with symptoms of hyperthyroidism lasting for approximately five months. Remarkably, she had developed pitting edema of her left leg four months before. She had gone through a conventional assessment, but the reason for the edema was not revealed. At presentation to the endocrinology clinic, the skin of both legs was of normal color and pitting edema on her left leg was of a diffuse nature and spread from her toes to two thirds of her leg. The skin surface of her left leg was smooth and had no elevations or discoloration, whereas her right leg appeared normal. Based on signs and symptoms of thyrotoxicosis and suppressed thyroid-stimulating hormone level (less than 0.001mIU/L, local reference of 0.4 to 4), treatment of 10mg of thiamazole three times a day was started. Additional blood tests revealed marked Graves’ hyperthyroidism with elevated free T4 and anti-thyroid receptor antibodies. Within a month, the free T4 level was normalized and the edema was completely cleared and never reappeared during the treatment course of 12 months.

**Conclusions:**

To the best of our knowledge, this is the first description of unilateral treatment-responsive leg edema as a manifestation of Graves’ hyperthyroidism. However, the pathophysiological mechanism underlying this case of edema remains unclear.

## Introduction

Graves’ disease may have various manifestations involving lower extremities. Graves’ dermopathy is a well-recognized entity that can appear as typical pretibial myxedema (57%) or plaques (21%) or rarely as nodular lesions or elephantiasis [1]. A less recognized, but relatively common, condition is bilateral pedal edema, which often is interpreted as a symptom of thyrotoxicosis-induced heart failure [2,3]. We describe a patient with Graves’ disease and unilateral leg edema, which had a clear temporal relationship with the course of thyrotoxicosis and cleared in parallel with a return of normal thyroid function.

## Case presentation

A 38-year-old Caucasian woman presented to the out-patient clinic with symptoms of hyperthyroidism lasting for around five months. She had lost approximately 3kg and had noticed irritability and mild hand tremor. On examination, signs of thyrotoxicosis were moderate: hand tremor, tachycardia (95 beats per minute), and warm skin were apparent. Her thyroid was smooth, had a slightly increased volume, and was not tender on palpation. There was no eye involvement except mild periorbital edema of her left eye. Remarkably, she had developed painless pitting edema of the left leg four months before. She had no history of trauma. She had gone through a conventional assessment, but the reason for the edema was not revealed. She did not report being on oral contraceptive pills and had had normal findings on Doppler ultrasound of her leg veins and normal D-dimer levels. At presentation to the endocrinology clinic, the skin of both legs was of normal color and the pitting edema on her left leg was of a diffuse nature and spread to two thirds of her leg. The skin surface was smooth and had no elevations or discoloration, whereas her right leg appeared normal. Based on signs and symptoms of thyrotoxicosis and suppressed thyroid-stimulating hormone level (less than 0.001mIU/L, local reference of 0.4 to 4), treatment of 10mg of thiamazole three times a day was started and additional blood tests were carried out. Blood tests revealed marked hyperthyroidism with elevated free T4 (75pmol/L, local reference of 10.3 to 24.5) and anti-thyroid receptor antibodies (13.5U/L, local reference of less than one and a half) but normal anti-thyroid peroxisomal antibodies (26IU/L, local reference of less than 35). A week later, the signs and symptoms of thyrotoxicosis were clearly improved; in parallel, the leg edema had markedly reduced. There was no pitting, and the maximal diameter of her left leg was close to that of her right leg (36.5 and 35cm, respectively). Within a month, the free T4 level was normalized (14pmol/L) and the edema had completely cleared. The edema never reappeared during the treatment course of 12 months.

## Discussion

Several descriptions of less frequent manifestations of Graves’ disease have been reported. In the context of the present case, two types of manifestations deserve attention. First, thyroid dermopathy may rarely manifest as diffuse edema of the leg (elephantiasic form). The possible pathogenetic mechanisms of the elephantiasic form have been discussed elsewhere [4]. However, we find it rather unlikely that our patient had thyroid dermopathy. First, the local finding was different from dermopathy: there was no evidence of any kind of skin discoloration, hyperpigmentation, or elevations. More importantly, rapid disappearance of long-lasting edema in parallel with normalization of thyroid function would be highly atypical of thyroid dermopathy. The complete remission of thyroid dermopathy has been reported to occur in 26% of patients but with an average time to remission of nearly nine years [5]. Several reports have described congestive heart failure with or without leg edema in patients with hyperthyroidism [2,3,6]. Also, pitting edema without heart failure has been reported by several authors in the context of hyperthyroidism [2,7-9]. In these reports, edema disappeared with the successful treatment of hyperthyroidism. We have found no description of treatment-responsive unilateral pitting leg edema associated with Graves’ disease in the literature. It is very clear that unilateral leg edema cannot be caused by heart failure. Thus, we speculate that, at least in some cases, local vascular mechanisms may be responsible in the case of leg edema and this type of edema may be unilateral on rare occasions. However, this hypothesis, in order to be proven, has to be tested in further studies looking at the possible pathophysiological mechanisms. Bilateral leg edema has been found in patients with various forms of hyperthyroidism. This is a first description of unilateral leg edema and so whether this manifestation is specific to Graves’ disease or may appear with other forms of hyperthyroidism is currently unclear.

## Conclusions

To the best of our knowledge, this is the first description of unilateral treatment-responsive leg edema as a manifestation of Graves’ hyperthyroidism. However, the pathophysiological mechanism underlying this case of edema remains unclear.

## Consent

Written informed consent was obtained from the patient for publication of this case report. A copy of the written consent is available for review by the Editor-in-Chief of this journal.

## Competing interests

The authors declare that they have no competing interests.

## Authors’ contributions

The authors contributed equally to drafting the manuscript and interpreting the case. VV handled the patient care. Both authors read and approved the final manuscript.
